# Hematological reactions in the inhabitants of the Arctic on a polar night and a polar day

**DOI:** 10.1002/iid3.323

**Published:** 2020-06-17

**Authors:** Svetlana N. Balashova, Anna V. Samodova, Liliya K. Dobrodeeva, Natalya K. Belisheva

**Affiliations:** ^1^ N. Laverov Federal Center for Integrated Arctic Research Arkhangelsk Russia; ^2^ Research Centre for Human Adaptation in the Arctic Branch of the Federal Research Centre "Kola Science Centre of the Russian Academy of Sciences" Apatity Russia

**Keywords:** Arctic, cortisol, cytokines, lymphocytes, monocytes, neutrophils, noradrenaline, photoperiod, reagins

## Abstract

**Purpose:**

The purpose of this study is to identify the features of hematological reactions in the inhabitants of the Arctic territory of the Kola Peninsula on a polar night and a polar day.

**Methods:**

The study included determining the hemogram, neutrograms, monocytograms, lymphocytograms, and phagocytic activity neutrophil granulocytes, enzyme immunoassay, flow cytometry.

**Results:**

It was established that during the polar night, there is an increase in the activity of migration of leukocytes from the marginal pool to the circulating pool, an increase in the intensity of phagocytosis by neutrophils, an increase in the concentrations of noradrenaline, cortisol, as well as an increase in hyperergic reactions involving immunoglobulin E and inhibitory processes due to an increase in interleukin‐10.

**Conclusion:**

A prolonged lack of sunlight causes a decrease in the reserve capacity for regulating homeostasis and forces the body to use proliferative reactions, which is reflected in the increase in stab neutrophils, large lymphocytes in the structure of the lymphocytogram and CD10+ lymphocytes. In winters, the frequency of neutropenia registration also increases to 13% of cases, the deficit of phagocytic activity of neutrophils; lymphopenia is recorded in 20% with T‐helper deficiency (37%). A part of the population probably has a relatively high degree of vulnerability to the action of natural environmental factors and is not able to completely restore the initial levels of the effectiveness of adaptation reactions in the summer. So at the end of the polar day in 8% of adults born in the north, neutropenia is recorded and in 21%—lymphopenia.

## Graphical abstract

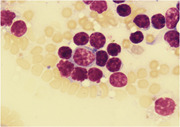

**Abbreviations**
CD4+: T helper cell
CD8+: cytotoxic T lymphocyte
CD10+: early B‐cell antigen
CIС: circulating immune complexes
IFN‐γ: interferon‐γ
IgA: immunoglobulin A
IgE: immunoglobulin E
IgG: immunoglobulin G
IgM: immunoglobulin M
IL‐10: interleukin‐10
IL‐12: interleukin‐12
TNF‐α: tumor necrosis factor‐α

## 1 INTRODUCTION

Photoperiods are the most stable and independent of human activity rhythms.^1^ The intensity of solar radiation and its spectral composition depends on solar activity, the height of the sun above the horizon, the mass of the atmosphere, and the presence of clouds.^2^ In the North, the annual total arrival of solar radiation is two times lower than that in the middle zone of the Russian Federation (112.71, respectively, and amounts to 299.59 kJ/cm^2^ per year). Its maximum level is reached in July (from 30.58 to 59.92 kJ/cm^2^) and the minimum falls on December (0.42 kJ/cm^2^). In the period from October to February, the negative radiation balance is on average 3.35 kJ/cm^2^. During this period, the low standing of the sun and the short duration of its radiance are noted; the largest—in July (334 hours), the smallest—in December and is only 1 hour. The period of absence of the influence of solar radiation is defined as biological darkness.^3^

A negative factor affecting the health of the inhabitants of the European North is the shortage of ultraviolet radiation. This territory is assigned to the zone with a significant deficit of the biologically active part of the solar spectrum of ultraviolet radiation. The circumpolar regions belong to the zone of prolonged insufficiency of ultraviolet radiation, which begins in October, and from the middle of November into the polar night, a period of biological darkness sets in. After the end of the polar night, the lack of ultraviolet radiation persists until April. It is known that infrared radiation is one of the most constant environmental factors continuously acting on the human body. The human body continuously absorbs and itself emits infrared rays; this heat transfer can vary significantly depending on the temperature of the human body and the environment.

Solar activity has a direct effect on the biological rhythms of all physiological functions.^4^, ^5^, ^6^

The influence of a change in photoperiodicity on the state of peripheral blood according to the literature is ambiguous. The most thoroughly studied is the change in the state of the erythron system.^7^, ^8^, ^9^, ^10^ There is evidence of an increase in the phagocytic activity of neutrophilic granulocytes in winters among residents of the middle zone of the Russian Federation.^11^

Deficiency of solar insolation is associated with a decrease in the activity of various enzyme systems, which causes changes in the mechanisms of regulation of the activity of metabolic processes and tissue respiration.^12^, ^13^, ^14^, ^15^

The influence of sunlight on hemodynamics and the cardiovascular system is especially great.^16^, ^17^, ^18^, ^19^, ^20^, ^21^ During the polar night, diastolic pressure rises.^22^, ^23^ When analyzing the phase structure of the cardiac cycle, 12.5% of patients examined during this period revealed myocardial hypodynamia syndrome and 25% showed an elongated isometric contraction phase.^24^ During this period, the minute volume of blood decreases,^24^, ^25^ the pulse can slow down,^22^, ^25^, ^26^, ^27^, ^28^ and the blood flow velocity decreases.^25^

In arterial blood, oxygen saturation does not differ from those in individuals living at the mid‐latitude level.^29^, ^30^ But the arteriovenous difference in oxygen significantly exceeds the norm, which indicates an increase in oxygen consumption^31^ and an increase in carbon dioxide in venous blood.^10^, ^32^

In winters, the blood content of residents of the Arctic territories is higher in cortisol.^23^, ^29^, ^33^, ^34^, ^35^

The period of the polar day is characterized by a predominance of the tone of the sympathetic part of the autonomic nervous system, in winters, there is a gradual transition to parasympathetic reactions.^22^, ^33^, ^36^

So, photoperiodicity—a change in the level of illumination—determines the chronobiological regulation of the functional activity of various body systems. The daily influence of solar insolation factors or their deficiency, as well as the long‐term repetition of such stressful situations, can cause a decrease in the effectiveness of regulatory mechanisms. Peripheral blood reactions to extreme factors appear first and quite objectively warn of possible stresses in regulatory mechanisms. Earlier, we established seasonal fluctuations in hemograms and parameters of the immune background in people born and permanently living in the Arctic in the Nenets Autonomous district and the Arkhangelsk region. It has been established that on a polar night, the frequency of detection of neutropenia, deficiency of phagocytic defence, the content of mature T‐lymphocytes and secretory immunoglobulin A (IgA) in the peripheral blood of the examined people increases.^37^, ^38^ In previous studies, it was found that people living in the Arctic Kola Peninsula showed signs of inhibition of switching immunoglobulin M (IgM) synthesis to immunoglobulin G, which significantly weakens the protective function of antibodies in tissues.^39^ The immune status is characterized by signs of a decrease in the reserve capacity for homeostasis regulation, which is manifested by a decrease in the activity of lymphocyte proliferation against the background of less significant concentrations of natural glycoproteins when compared with residents of the Nenets Autonomous Okrug.^40^

This study presents the results of a comparative study of hemograms and immune status in residents of the polar region of the Murmansk region.

The Revda settlement of (67° 56′ 13″ middle latitude) and Lovozero village of (68° 02′ 00″ middle latitude) Lovozersky district of the Murmansk region of the Russian Federation are located beyond the Arctic Circle in the central part of the Kola Peninsula. It is known that this territory belongs to a very unfavorable zone with intense environmental and climatic conditions affecting people and critical stress of adaptation systems,^41^ and according to living conditions—to an extremely uncomfortable zone.^42^, ^43^ Biological twilight and polar night, when a negative radiation balance is recorded, last from October to February; polar night lasts 25 days from 10 December to 3 January; the polar day lasts 52 days from 27 May to 17 July.

The problem of human adaptation to a pronounced change in the photoperiod is determined by a change in physiological reactions, including the endocrine and immune systems. But the issue of ensuring adaptation to contrast photoperiodic in extreme situations remains unresolved. In this regard, the aim of the study is to identify the features of hematological reactions in the inhabitants of the Kola Peninsula on a polar night and a polar day.

## 2 MATERIALS AND METHODS

The object of study were the inhabitants of the Arctic territory, aged 21‐ to 60‐years old, living in the Arctic Revda settlement and village Lovozero, Lovozero district, Murmansk region of the Russian Federation. The study includes the results of a survey of 249 healthy people: 131 people were examined during the polar night and 118 people on a polar day.

All studies were conducted with the consent of the volunteers and in accordance with the requirements of the Helsinki Declaration of the World Medical Association. Ethical principles for conducting medical research involving a person as a subject (1964, as amended and supplemented from 2013).

For the study, peripheral venous blood was taken from the ulnar vein in the morning on an empty stomach. The number of cells of the leukogram, monocytogram, lymphocytogram, and neutrogram were counted in blood smears stained by the Romanovsky–Giemsa method; monocytogram was determined by the method of Grigorova (1956), lymphocytogram—according to the method of Kassirsky (1970), neutrogram—according to the method of Todorov (1968). The phagocytic activity of neutrophils was studied using the test kit from Reacomplex (Chita, Russia). Isolation of mononuclear cells from peripheral blood was performed according to the method of Boymn (1976), phenotyping of lymphocytes in an indirect immunoperoxidase reaction using monoclonal antibodies (MedBioSpektr; Sorbent, Moscow, Russia), and flow cytometry using an Epics XL apparatus from Beckman Coulter with Immunotech a Beckman Coulter Company reagents (France).

In blood serum by the solid‐phase method, enzyme‐linked immunosorbent assay on an automatic enzyme‐linked immunosorbent analyzer Evolis (Bio‐Rad, Germany) with the appropriate reagents determined the content of cytokine interleukin‐10 (IL‐10; Seramnum Diagnostica, Germany), noradrenaline (IBL, Germany), and cortisol (DBC, Canada). The concentration of circulating immune complexes (CIK) was determined by the standard precipitation method using 3.5%, 4.0%, and 7.5% PEG‐6000 in blood serum. The reaction was evaluated on Evolis.

Statistical processing of the obtained data was carried out using the Statistica 10.0 software package (StatSoft) with a determination of average values and presented as the arithmetic mean ± error of the mean (M ± m), the significance of differences was evaluated using Student's *t* test. The results of nonparametric processing methods are presented as the median (Me) and interquartile range in the form of 25 and 75 percentiles. The statistical significance of the differences was determined using the nonparametric Mann–Whitney test. The critical level of significance (*P*) in the study was taken equal to .05.

People were examined on 3 to 5 December and 18 to 20 June, that is, during periods of maximum severity of the polar night and the greatest duration of the polar day. Neutrophilic leukocytosis was detected with a neutrophilic granulocyte count more than 5.5 × 10^9^ cells/L, a neutrophilic granulocyte count deficit (neutropenia) less than 2.0 × 10^9^ cells/L, lymphocytosis more than 3.0 × 10^9^ cells/L, lymphopenia less than 1.5 × 10^9^ cells/L and monocytosis—0.6 or more × 10^9^ cells/L.

## 3 RESULTS AND DISCUSSION

**Figure 1:**
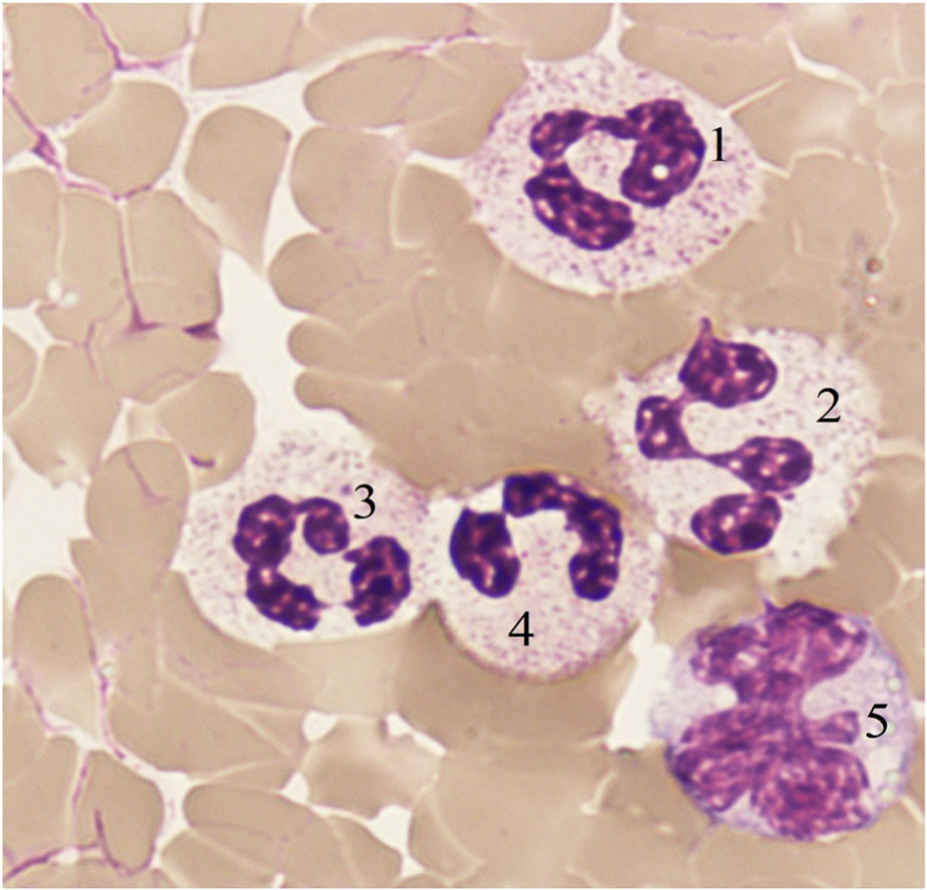
Neutrophilic leukocytosis. Peripheral venous blood. Coloring according to Romanovsky–Giemsa. ×1000. 1, 2, 3, 4: neutrophilic white blood cells, 5: monocyte

During the polar night, the frequency of registration of increased concentrations of circulating neutrophilic granulocytes is higher (22.90% ± 0.36% and 2.63% ± 0.42% of the subjects, respectively, Figure 1). This neutrophilic leukocytosis is accompanied by a “left shift” of 60.00% ± 2.57% with an increase in the concentration of stab neutrophilic granulocytes (from 0.43 ± 0.12 to 0.70 ± 0.13 × 10^9^ cells/L; *P* = .028). Activation of the proliferation of neutrophilic granulocytes is accompanied by an increase in their apoptosis activity with an increase in the composition of cells with five or more nuclear segments (0.03 ± 0.01 and 0.29 ± 0.07 × 10^9^ cells/L, respectively; *P* = .003).

Along with an increase in the concentration of circulating neutrophilic granulocytes in winters, neutropenia was detected in 12.98% ± 0.27% of the examined individuals; during the polar day, neutropenia is set at 7.89% ± 0.73%. In cases of neutropenia, a deficit of phagocytic activity of these cells was recorded; the frequency of registration of a deficit of phagocytic protection in winter was noticeably higher than in summer (45.05% ± 0.51% and 36.84% ± 1.40%, respectively).

In winters, the rate of monocytosis is higher than in summers (45.04% ± 0.51% and 21.05% ± 1.20%, respectively). The increase in the concentration of circulating monocytes is mainly provided by the redistribution from the marginal to the circulating pool due to mature monocytes (0.59 ± 0.08 and 0.18 ± 0.02 × 10^9^ cells/L, respectively; *P* < .001) without signs of proliferative activity according to the analysis of monocytograms. One of the most important functions of monocytes in the phagocytosis of immune complexes circulating in the bloodstream by receptor endocytosis.^44^ But despite the increase in the blood monocyte content in the winter, the frequency of registration of elevated concentrations of CIK is much higher than on a polar day: CIK IgA 60.26% ± 0.59%, CIK IgA 75.64% ± 0.66%; on a polar day, 27.03% ± 1.36% and 48.65% ± 1.83%, respectively. Even in the average results, the concentrations of CIK in the blood during the polar night were significantly higher (CIK IgA and IgM: 4.48 ± 0.29 and 5.41 ± 0.31 g/L vs 2.56 ± 0.30 and 3.59 ± 0.45 g/L; *P* < .001).

**Figure 2:**
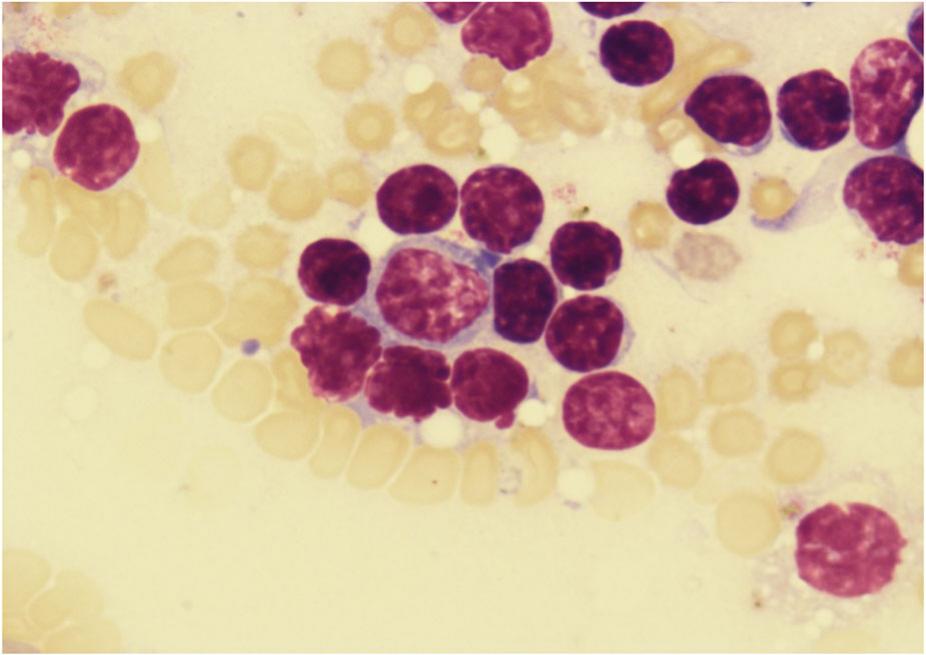
Lymphocytosis. Peripheral venous blood. Coloring according to Romanovsky–Giemsa. ×1000

Quite often, lymphopenia was recorded at practically the same level both in winters and summers (19.85% ± 0.34% and 21.05% ± 1.20%); the detection rate of lymphocytosis during the polar night was 1.5 times higher (29.01% ± 0.41% and 18.42% ± 1.12%; Figure 2). There is reason to believe that lymphocytosis on a polar night is ensured by lymphoproliferation, since the content of large lymphocytes in the lymphocytogram is much higher even in average results (0.33 ± 0.04 vs 0.14 ± 0.02 × 10^9^ cells/L; *P* < .001). Signs of increased proliferative activity of lymphocytes with an increase in the content of CD10+ phenotype in the peripheral blood, potentially capable of proliferation, were detected in winters at 35.92% ± 0.46% and 37.50% ± 1.60% in summers. During the polar night, an increase in the content of CD8+ cytotoxic phenotypes was recorded at 37.50% ± 0.47%; in cases of lymphocytosis, increased concentrations of CD8+ were found at 84.38% ± 3.52%. Against the background of increased activity of cell‐mediated cytotoxicity, a very high level of deficiency of T‐helper cells was established in winter and summer periods (37.50% ± 0.47% and 37.04% ± 1.59%).

So, during the polar night, the content of neutrophilic leukocytes (22.90% ± 0.36%), monocytes (45.04% ± 0.51%), and lymphocytes (29.01% ± 0.41%) circulating in venous blood is higher. The increase in white blood cell count is due to the activation of proliferative processes of the corresponding cell sprouts.

An increase in the content of neutrophilic granulocytes is not accompanied by an increase in % of active phagocytes, the intensity of phagocytosis in terms of the phagocytic number increases (5.51 ± 0.11 vs 3.92 ± 0.14 units/cell; *P* < .001). In winters, the frequency of neutropenia registration also increases (from 7.89% ± 0.73% to 12.98% ± 0.27%) and the deficit of phagocytic activity of neutrophils from 36.84% ± 1.40% to 45.05% ± 0.51% in these cases. It is known that neutrophilic granulocytes, making up the bulk of circulating leukocytes, not only phagocytize, are antigen‐reactive cells, perform antibody‐dependent cytolysis of affected, transformed and old cells, and are sources of a huge number of primary and secondary mediators of inflammatory reactions and all known cytokines.^45^, ^46^, ^47^, ^48^ Deficiency of the content of these cells creates serious problems in the regulation and provision of almost all adaptive reactions.

Along with lymphocytosis during the polar night, the incidence of lymphopenia increases. Given the role of lymphocytes in the implementation of immune responses, the high prevalence of T‐helper deficiency, reaching 30% to 37%, we can say with alarm about the risk of the formation of secondary environmentally dependent immunodeficiency in a person living in the Arctic. It is possible that a certain contribution to this is made by factors associated with a lack of sunlight. In the North, from October to February, the sun is low and its duration is short; the highest is in July (334 hours), the lowest is in December and is only 1 hour. The period of absence of the influence of solar radiation is defined as biological darkness.^3^ Adverse climatic factors include the lack of total solar radiation. The total radiation in the North in September is two times, and in October, five times less than in the middle zone of the Russian Federation, and in the next four months (November‐February), there is practically no natural radiation of the sun in the north. A negative factor affecting the health of the inhabitants of the European North is the shortage of ultraviolet radiation.

Reactions with a change in the concentration of circulating leukocytes are provided primarily by changes in hemodynamics. It is known that a dynamic equilibrium is established between circulating and parietal cells, which is constantly shifting toward an increase or decrease in both its components.^49^ Migration and perfusion of cells are provided by a significant slowdown in blood flow in the capillary network of the bloodstream. In this case, it becomes possible for the cell to adhere to the capillary wall with its subsequent exit beyond the vascular bed.^50^, ^51^, ^52^

Changing the ratio of circulating and parietal pools are the main signal for a hemodynamic reaction. The microvasculature is a system of transport blood flow, its functional state varies depending on the state of the tissues provided by the blood in this section of the blood flow.^53^, ^54^, ^55^, ^56^, ^57^, ^58^, ^59^, ^60^ The functional state of the microvasculature is ensured by numerous regulatory mechanisms: endothelial origin with the secretion of nitric oxide and vasoconstrictor endothelin‐1, neurogenic sympathetic activity.^61^ Hemodynamic reactions are provided by catecholamines and prolonged by cortisol.^62^, ^63^, ^64^ Indeed, during the polar night, in individuals with a deficiency of neutrophilic granulocyte and lymphocyte counts, an increased content of noradrenalin (10.92% ± 0.25% and 0.85% ± 0.08%) and cortisol (20.00% ± 0.34% and 9.53 ± 0.26%), respectively. Violation of the ratio of catecholamines and cortisol causes imperfection of regulatory mechanisms and is characteristic of a state of readiness for stress, a state of allostasis. The immunosuppressive properties of cortisol are widely known.^65^ There is also a tendency to increase the content of this hormone in the inhabitants of the northern territories.^23^, ^29^, ^33^, ^34^ A relatively constant increase in the content of cortisol causes the formation of an immunodeficiency state. Among persons with deficiency of T‐helpers, the frequency of recording high concentrations of cortisol in the blood is markedly higher (85.71% ± 3.57%). It is known that the content of IL‐10 is associated with immunosuppression and increases in chronic pathology.^66^, ^67^, ^68^, ^69^ Activation of IL‐10 production can be initiated by an increase in blood catecholamines by suppressing the functional activity of T‐helpers and their production of pro‐inflammatory cytokines such as interferon‐γ, tumor necrosis factor‐α, and IL‐12.^70^ In such immunosuppression conditions, it is justified to activate an alternative class of immunoglobulin E (IgE), which enhances the effectiveness of immune defence by involving the powerful cytolytic potential of eosinophils and basophils. Indeed, with a low level of light mode, the immune response in Arctic residents is accompanied by increased concentrations of IgE (25.57% ± 0.38% and 16.36% ± 0.34%) and IL‐10 in the blood (10.23% ± 0.24% and 2.04% ± 0.12%).

## 4 CONCLUSION

It seems that only those who have a certain adaptive reserve can fully adapt to the sharply changed polar night and polar day. The increased need for leukocytes in tissues in conditions of deficiency of sunlight can be compensated by an increase in the metabolic activity of leukocytes (increased activity of leukocyte migration from the marginal pool, the intensity of phagocytosis by neutrophils), a change in the hemodynamics of the microvasculature by catecholamines and the release of cells from the depot. Long‐term exposure to the factor leads to a reduction in the reserve capacity for regulating homeostasis and forces the body to use proliferative reactions.

A part of the population probably has a relatively high degree of vulnerability to the action of natural environmental factors and is not able to completely restore the initial levels of the effectiveness of adaptation reactions in the summer. So at the end of the polar day in 8% of adults born in the north, neutropenia is recorded and in 21%—lymphopenia.

Hyperergic reactions involving IgE and inhibitory processes due to IL‐10 are, in our opinion, the criteria for an unfavorable state of adaptive mechanisms. Elevated concentrations of IgE during the polar night are found in 26% in individuals who have no history of allergy; in summer, the frequency of their registration decreases to 16%. IL‐10 inhibition was detected in December at 10%, and in summer at 2%.

## CONFLICT OF INTERESTS

The authors declare that there are no conflict of interests.

## References

[1] Wehr TA . Photoperiodism in humans and other primates: evidence and implications. J Biol Rhythms. 2001;16(4):348‐364. 10.1177/074873001129002060 11506380

[2] Kalitin NN . Sun beams. Publ House USSR Acad Sci. 1947:112.

[3] Galanin NF . The problem of compensation for natural ultraviolet deficiency in the North. Hyg Sanit. 1955;5:54‐55. 14391583

[4] Vladimirsky BM . Biological rhythms and solar activity Problems of space biology. Moscow: Nauka; 1980:289‐315.

[5] Komarov FI , Rapoport SI , Breus TK , Ivanova SV . Solar‐ionospheric communications of biorhythms and some issues of internal medicine: methodological aspects. Ther Arch. 1985;57(3):149‐153. 3890256

[6] Komarov FI , Breus TK , Rapoport SI , Musin MM , Setov IV . Heliogeophysical factors and their effect on cyclic processes in the biosphere. Res Sci Technol Ser Med Geogr. 1989;8:175.

[7] Marachev AG . Morphophysiological indicators of red blood in the inhabitants of the Far North. Hum Physiol. 1977;2(1):106‐111.

[8] Vergunova ZI . Comparative evaluation of changes in red blood with exo‐ and endogenous hypoxemia in the age aspect. Simferopol. 1978:25.

[9] Degteva GN . The state of erythropoiesis is normal and with pathology among residents of the European North of the USSR. Med Sci. 1986:22.

[10] Marachev AG . Morphofunctional fundamentals of adaptation and pathology of the lungs, heart and red blood of a person in the Far North. Med Sci. 1980:60.

[11] Fedorova MZ . Functional properties and reactivity of blood leukocytes in altered conditions of the body caused by factors of various nature. Yaroslavl. 2002:294.

[12] Gnevyshev MN . Heliophysical foundations of solar‐biological bonds. In the book: the influence of geophysical and meteorological factors on the vital functions of a body. Novosibirsk. 1978:15‐24.

[13] Benevolensky BN , Voskresensky AD . Heliobiological researchs: current status and prospects. Bull USSR Acad Sci. 1980;10:54‐65.

[14] Novikova KF , Byakov VM , Mikheev YUP , et al. Adaptation issues and solar activity Problems of space biology. The effect of solar activity on the biosphere. 43 Moscow: Nauka; 1982:9‐46.

[15] Assman D . Sensitivity to weather: translation from German. Hydrometeoizdat. 1966:247.

[16] Kuberger MB . Guidelines for clinical electrocardiography of children. Medicine. 1983:368.

[17] Bardov VG , Gabovich RD , Nikberg II . On the problem of the relationship of the frequency of occurrence of hypertensive crises with a change in solar activity and magnetic field strength. Hyg Sanit. 1977;8:111‐115. 590786

[18] Kuzmenko VA , Buluev AB . Changes in blood pressure during static operation depending on the time of day and the degree of perturbation of the Earth's magnetic field. Hum Physiol. 1983;6:892‐895.

[19] Ryzhikov GV , Dzhebrailova TD . The effect of geomagnetic disturbances on the state of cardiovascular functions in athletes. Hum Physiol. 1984;10(4):640‐646. 6544722

[20] Chegodar AYA . The influence of electromagnetic fields of low frequency and various tension on the cardiovascular system of animals. Simferopol. 1972:17.

[21] Chibisov SM . Change in the functional state of the heart in various phases of the solar cycle V All‐Union Symposium – Ecological and physiological problems of adaptation. 1988:251‐252.

[22] Kandror IS . Essays on the physiology and hygiene of man in the Far North. Medicine. 1968:280.

[23] Neverova NP . The state of vegetative functions in healthy people in the Far North. Novosibirsk. 1972:39.

[24] Turchinsky VI . Some biomedical aspects of the adaptation of Taimyr natives. In the book: biomedical aspects of adaptation processes. Novosibirsk. 1975:209‐219.

[25] Deryapa NR , Matusov AL , Ryabinin IF . Man in Antarctica. Medicine. 1975:183.

[26] Pavlovsky OM . The biological age of a person. Publ House Moscow State Univ. 1987:280.

[27] Wilson О . Нuman adaptation to life in Antarctica In: van Mieghem J, van Hague Oye P, eds. Biogeography and Ecology in Antarctica, 1965:690‐752.

[28] Deryapa NR . The nature of Antarctica and the acclimatization of man. ML. 1965:155.

[29] Bobrov NI , Lomov OP , Tikhomirov VP . Physiological and hygienic aspects of human acclimatization in the North. Medicine. 1979:184.

[30] Kaznacheev VP , Egunova EE , Kulikov VYu . Some features of oxygen metabolism in the process of human adaptation to high latitudes. Scientific and technological progress and circumpolar medicine. Novosibirsk. 1978:126‐127.

[31] Tenditnaya LV . Some indicators of seasonal changes in gas exchange and basic metabolism in children – indigenous people of the Far North. Physiology and pathology of human adaptation in the Far North. Novosibirsk. 1977:99‐103.

[32] Ilyin VA . Features of peripheral blood circulation in healthy children (8‐15 years old), natives of various altitude zones. Frunze. 1982:22.

[33] Neverova NP . The functional state of the adrenal glands during acclimatization in the Far North. Acclimatization Man Polar Regions. 1969:52‐53.

[34] Weitzman ED , deGraaf AS , Sassin JF , et al. Seasonal patterns of sleep stages and secretion of cortisol and growth hormone during 24 hour periods in Northern Norway. Acta Endocrinol. 1975;78:65‐76. 10.1530/acta.0.0780065 163564

[35] Tkachev AV , Ramenskaya EB . Ecological and physiological features of the pituitary gland‐adrenal cortex‐thyroid gland Endocrine system and metabolism in humans in the North. Syktyvkar: KSC Ural Branch of RAS; 1992:15‐44.

[36] Pinkovskaya EY . Features of changes in the autonomic nervous system in the process of acclimatization in the North. Features of the pathology of the autonomic nervous system in the North. Arkhangelsk. 1972:95‐97.

[37] Dobrodeeva LK , Zhilina LP . Immunological reactivity, the state of health of the population of the Arkhangelsk region. Ekaterinburg: Ural Branch of RAS; 2004:229.

[38] Dobrodeeva LK , Senkova LV , Moskovskaya NB . Environmentally dependent changes in immunity in the North. In the book: physiological patterns of hormonal, metabolic, immunological changes in the human body in the European North. Syktyvkar. 1997:97‐116.

[39] Samodova AV , Tsypysheva OB . The ratio of the extracellular pool of receptors and the level of immune responses in people living in the Arctic. Hum Ecol. 2015;12:21‐27.

[40] Patrakeeva VP , Balashova SN , Samodova AV . The proliferative activity of peripheral blood cells in Arctic residents. Natural resources and integrated development of the coastal regions of the Arctic zone: collection of scientific works. Repl ed Doctor of Economics, prof. V.I. Pavlenko. 2016:374‐379.

[41] Vinogradova VV . Natural‐climatic and bioclimatic living conditions of the population of the Murmansk region. Proc RAS. Geography Ser. 2015;6:90‐99.

[42] Selin VS , Vasiliev VV , Shirokova LN . Russian Arctic: geography, economics, regionalization. Apatity: KSC RAS; 2011:203.

[43] Selin VS , Vasiliev VV . The method of integrated environmental zoning and the allocation of the southern border of the Russian Arctic. Bull Kola Sci Cent Russ Acad Sci. 2014;1(16):64‐71.

[44] Fridman WH . Fc receptors and immunoglobulin binding factors. FASEB J. 1991;5:2684‐2690. 1916092 10.1096/fasebj.5.12.1916092

[45] Ottonello L , Epstein AL , Mancini M , Dapino P , Dallegri F . Monoclonal LYM‐1 antibody‐dependent cytolysis by human neutrophils exposed to GM‐CSF: auto‐regulation of target cell attack by cathepsin G. J Leukoc Biol. 2003;75(1):99‐105. 10.1189/jlb.0403133 14525961

[46] Vorobyeva NV . NADPH‐neutrophil oxidases and diseases associated with its dysfunction. Immunology. 2013;34(4):227‐233.

[47] Cascao R , Rosario HS , Fonseca JE . Neutrophils: warriors and commanders in immune mediated inflammatory diseases. Acta Reumatol Port. 2009;34:313‐326. 19727044

[48] Lugovskaya SA , Kozinets GI . Hierarchy of hematopoietic cells: kinetics, structure and functions (I part) (lecture). Clin Lab Diagn. 2009;5:21‐36. 19537338

[49] Fedorov NA, ed. Normal blood formation and its regulation. Medicine; 1976:543.

[50] Ambrus CM , Ambrus JL . Regulation of the leukocyte level. Ann NY Acad Sci. 1959;77:445‐486. 10.1111/j.1749-6632.1959.tb36920.x 13793132

[51] Carper HA , Hoffman PL . The intravascular survival of transfused canine Pelger‐Huet neutrophils and eosinophils. Blood. 1966;27:739‐743. 10.1182/blood.V27.5.739.739 5936828

[52] Meuret G , Fliedner TM . Neutrophil and monocyte kinetics in a case of cyclic neutropenia. Blood. 1974;43:565‐571. 10.1182/blood.V43.4.565.565 4816844

[53] Barkhatov IV . The use of laser Doppler flowmetry to assess violations of the human blood microcirculation system. Kazan Med J. 2014;95(1):63‐69.

[54] Choi CM , Bennett RG . Laser Dopplers to determine cutaneous blood flow. Dermatol Surg. 2003;29:272‐280. 10.1046/j.1524-4725.2003.29042.x 12614422

[55] Kozlov VI , Sokolov VG . Study of blood flow fluctuations in the microcirculation system Materials of the Second All‐Russian Symposium – The use of laser Doppler flowmetry in medical practice. 1998:8‐13.

[56] Krechina EK , Kozlov VI , Maslova VV . Microcirculation in periodontal gum tissues. GEOTAR‐Media. 2007:80.

[57] Krupatkin AI . New possibilities for assessing the innervation of skin microvasculature using spectral analysis of microhemodynamic oscillations. Reg Blood Circ Microc. 2004;4:52‐59.

[58] Timerbulatova VM, ed. The use of laser Doppler flowmetry in endoscopy and endosurgery for emergency diseases of the abdominal organs. Moscow, Russia: MEDpress‐Inform; 2006:107.

[59] Bergstrand S , Lindberg LG , Ek AC , Lindén M , Lindgren M . Blood flow measurement at different depths using photoplethysmography and laser Doppler techniques. Skin Res Technol. 2009;15:139‐147. 10.1111/j.1600-0846.2008.00337.x 19622122

[60] Humeau A , Steenbergen W , Nilsson H , Stromberg T . Laser Doppler perfusion monitoring and imaging: novel approaches. Med Bio Eng Comput. 2007;45:421‐435. 10.1007/s11517-007-0170-5 17340155

[61] Buckton KE , Brown WMС , Smith PG . Lymphocyte survival in men treated with X‐rays for ankylosing spondylitis. Nature. 1967;214(87):470‐473. 10.1038/214470a0 6032868

[62] Chertkov IL , Friedenstein AY . Cellular basis of hematopoiesis. Moscow, Russia: Medicine; 1977:272.

[63] Chrousos GP . The HPA axis and stress response. Endocr Res. 2000;26(4):513‐514. 10.3109/07435800009048562 11196421

[64] Ostrander MM , Ulrich‐Lai YM , Choi DC , Richtand NM , Herman JP . Hypoactivity of the hypothalamo‐pituitary‐adrenocortical axis during recovery from chronic variable stress. Endocrinology. 2006;147(4):2008‐2017. 10.1210/en.2005-1041 16396985 PMC1815381

[65] Bagby GC , Gabourel JD , Linman JW . Glucocorticoid therapy in the preleukemia syndrome (hemopoietic dysplasia): identification of responsive patients using in vitro techniques. Ann Intern Med. 1980;92(1):55‐58. 7350872 10.7326/0003-4819-92-1-55

[66] Bergmann C , Strauss L , Zeidler R , Lang S , Whiteside TL . Expansion and characteristics of human T regulatory type 1 cells in co‐culture simulating tumor microenvironment. Cancer Immunol Immunother. 2007;56(9):1428‐1442. 10.1007/s00262-007-0280-9 PMC11031003 17265021

[67] Du C , Wang Yu . The immunoregulatory mechanisms of carcinoma for its survival and development. J Еxp Clin Cancer Res. 2011;30:12 10.1186/1756-9966-30-12 PMC3031251 21255410

[68] Rabinоvich GA , Ilarregui JM . Conveying glycan information into T cell homeostatic programs: a challenging role for galectin‐1 in inflammatory and tumor microenvironments. Immunol Rev. 2009;230(1):144‐159. 10.1111/j.1600-065X.2009.00787.x 19594634

[69] Phiеler J , Chung KJ , Chatzigeorgiou A , et al. The complement anaphylatoxin C5a receptor contributes to obese adipose tissue inflammation and insulin resistance. J Immunol. 2013;191(8):4367‐4374. 10.4049/jimmunol.1300038 24043887 PMC3817864

[70] Sherstoboev EY , Babenko AP . Modulation of cytokine production by adrenomimetics against the background of stress and antigen exposure. Cytokines Inflamm. 2007;6(3):40‐43.

